# Metastatic Adenocarcinoma of Unknown Origin Presenting as Small Bowel Perforation: A Case Report and Literature Review

**DOI:** 10.1177/2324709615577415

**Published:** 2015-03-23

**Authors:** Samir Alkabie, Brian Bello, Roberto F. Martinez, W. Peter Geis, Michael S. Ballo

**Affiliations:** 1 Northwest Hospital, Randallstown, MD, USA; 2 Saba University School of Medicine, Devens, MA, USA; 3 Sinai Hospital, Baltimore, MD, USA

**Keywords:** occult primary, cancer of unknown primary site, small bowel perforation, pulmonary primary, metastatic adenocarcinoma, diagnosis, immunohistochemistry

## Abstract

Metastatic malignant tumors that originate from occult primaries are defined as “cancers of unknown origin.” We herein present the case of a 59-year-old man who presented with small bowel perforation secondary to metastatic adenocarcinoma of an unknown primary site. Imaging exhibited two pulmonary nodules, neither of which was dominant, along with mediastinal and retroperitoneal lymphadenopathy. Immunohistochemical profiling of the small bowel biopsy specimens revealed the tumor was most likely pulmonary in origin.

## Introduction

Cancer of unknown primary site (CUP) is a heterogeneous group of cancers, comprising 3% to 5% of all malignancies,^1^ the vast majority of which are adenocarcinoma (80% to 90%).^2,3^ It is referred to as an “orphan” disease, diagnosed on histologic detection of metastases while the anatomical site of origin remains elusive after initial workup.^3^ Its clinical course is aggressive, characterized by a short preclinical history, early dissemination, resistance to chemotherapy, and overall dismal prognosis with a median life expectancy of 6 to 9 months.^3-5^ Smoking is the most important risk factor for developing occult malignancy,^3^ and the most common primary sites are pancreas and lung.^4-6^

The National Comprehensive Cancer Network published their guidelines in 2014 concerning the initial evaluation, workup, and pathological diagnosis of occult primary cancers.^7^ The evaluation and workup of suspected metastatic malignancies requires a thorough history and complete physical exam; complete blood count; comprehensive metabolic panel; computerized tomography (CT) of chest, abdomen, and pelvis; hemoccult test; and symptom-directed endoscopy.^7^ Pathologic diagnosis is achieved by subjecting biopsy specimens to immunohistochemistry and gene expression profiling, which predicts the most likely tissue of origin.^7^ Tissue of origin identification and subsequent tissue-specific therapy may improve patient outcomes.^8^ A recent large prospective study demonstrated a survival advantage in patients treated with site-specific therapy guided by molecular gene expression profiling compared with empiric regimens.^9^ Yet responses and survival in CUP are generally poor.^1^ We report on a patient presenting with an acute abdomen who was found to have a small bowel perforation secondary to metastatic disease from an unknown primary site.

## Case Report

A 59-year-old African American man presented to the emergency department in moderate distress, complaining of severe, diffuse abdominal pain associated with nausea, vomiting, chills, diaphoresis, and constipation. The pain had started 1 week prior as a dull ache, worsened for several days, and became severe with stabbing abdominal pain the day of admission. Family history was positive for cancer in his father and mother as well as 3 siblings, but he was unaware of their diagnoses, except that one brother had prostate and “bowel cancer.” He had a 12 pack-year smoking history and was a current smoker. Physical examination demonstrated he was diffusely tender in his abdomen and had involuntary guarding consistent with peritonitis.

**Figure 1.**
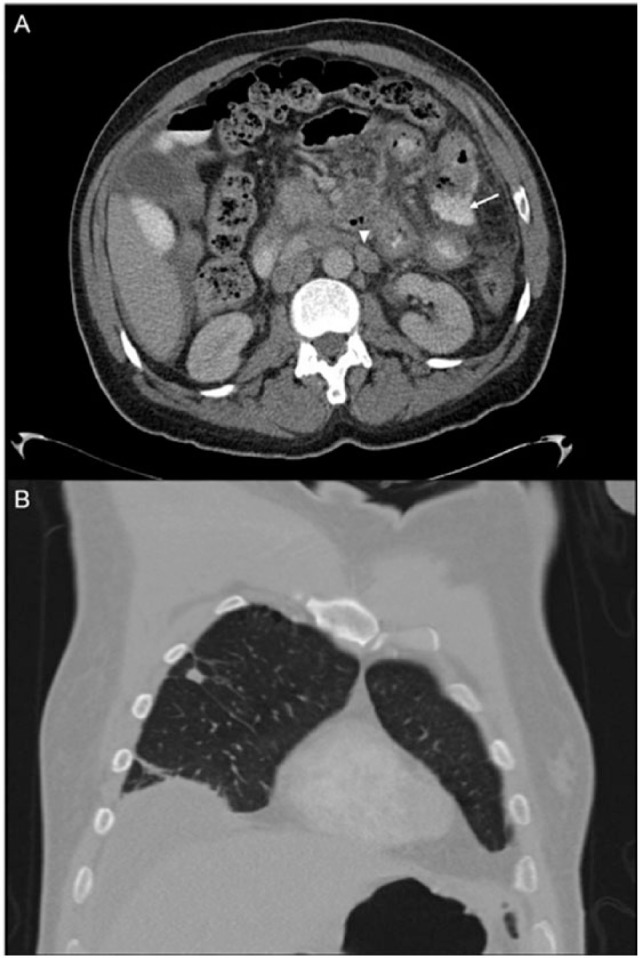
Computed tomography scans showing (A) extravasated oral contrast (arrow) and an enlarged periaortic lymph node (arrow head) and (B) a 1.1 cm noncalcified, nonspiculated pulmonary nodule of the right upper lobe.

Vitals were as follows: temperature 36.8°C, pulse 138 beats/minute, respiratory rate 18 breaths/minute, and blood pressure 118/66 mm Hg. Laboratory evaluation showed a total white blood cell count of 6.55 × 10^3^/mm^3^, 24% neutrophils, 52% bands, 5% lymphocytes, albumin 3.1 g/dL, sodium 132 mmol/L, potassium 3.1 mmol/L, chloride 93 mmol/L, bicarbonate 30 mmol/L, blood urea nitrogen 26 mg/dL, and serum creatinine 1.3 mg/dL, and the rest of the values were normal. CT imaging of the abdomen demonstrated free intraperitoneal air and small bowel thickening, as well as intraperitoneal extravasation of contrast into the left upper quadrant (Figure 1A), indicating bowel perforation. CT imaging also showed lymphadenopathy of the chest and abdomen and 2 pulmonary nodules in the right upper lobe measuring up to 1.1 cm in maximal diameter (Figure 1B).

Exploratory laparotomy exposed a small bowel perforation related to a full-thickness mass of the jejunum. In addition, there were multiple palpable intraluminal masses approximately every 10 cm throughout the jejunum. Diffuse retroperitoneal, pelvic, and mesenteric lymphadenopathy was appreciated. Two areas of small bowel were resected, one segment where the bowel had perforated and a second that was nearly perforated. The serosa of the second area was thin and friable, consistent with impending perforation.

**Figure 2.**
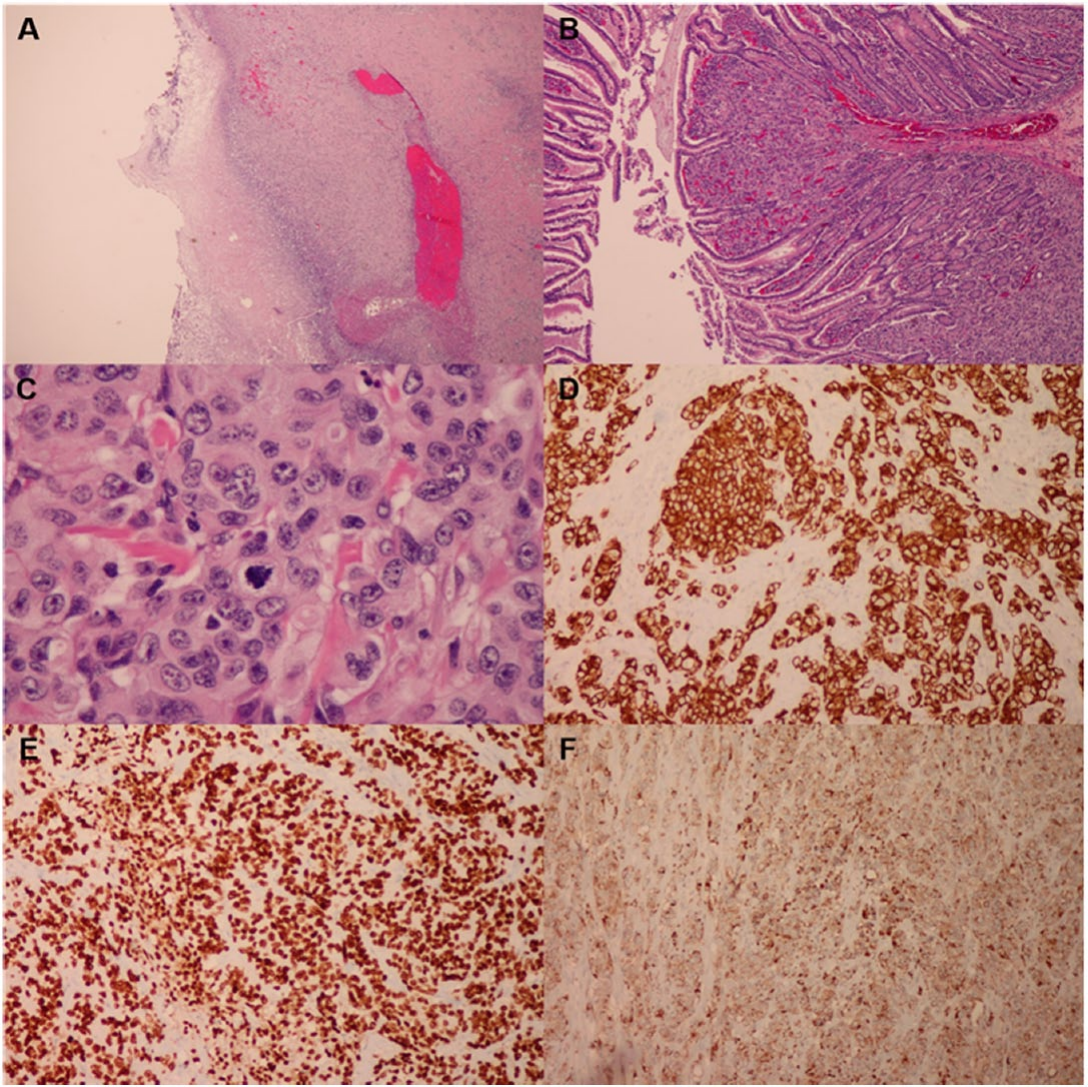
Jejunum biopsy. Hematoxylin–eosin staining (A-C) and immunohistochemistry (D-F). (A) Low-power view of transmural wall involvement with tumor cell infiltration and necrosis. (B) Low-power small intestine villi infiltrated with tumor cells. (C) High-power view of tumor cells and mitotic figure (center). (D) CK7 cytoplasmic immunostaining. (E) TTF-1 nuclear immunostaining. (F) Napsin A, granular cytoplasmic immunostaining.

Histopathology of the resected specimens revealed metastatic adenocarcinoma with transmural involvement of the small intestine, with mucosal ulceration, necrosis, and perforation (Figure 2A-C). The lymphatic vessels were markedly dilated and engorged with malignant cells. Tumor was present at proximal, distal, and mesenteric resection margins. There were areas of prominent serosal inflammation with exudates, consistent with peritonitis. The tumor cells were immunoreactive for cytokeratin 7 (CK7; Figure 2D), thyroid transcription factor-1 (TTF-1; Figure 2E), and napsin A (Figure 2F) and were negative for CD20, CDX2, P63, chromogranin, synaptophysin, and CD56. A special stain for mucin was positive. Altogether, the biopsy stainings were consistent with a pathological diagnosis of metastatic adenocarcinoma from a pulmonary primary.

His postoperative hospital course was unremarkable. He was discharged on postoperative day 5 with home care. At home, he had occasional fatigue, night sweats, insomnia, poor appetite, nausea, vomiting, as well as weight loss (4 kg in 2 weeks and a cumulative 23 kg loss from his normal set point) and alternating diarrhea and constipation. He met with medical oncology who ordered further imaging studies, bronchoscopy, molecular testing of the tumor, and chemotherapy. He was readmitted 1 month after surgery for dehydration and weakness with a deteriorating functional status. After aggressive fluid resuscitation and electrolyte repletion he was discharged, but he was readmitted 2 weeks later with severe and worsening abdominal pain. He was very ill appearing, with leukocytosis, critical lactic acidosis, and acute renal failure. CT of the abdomen confirmed free air and the presence of another small bowel perforation with a large amount of fluid in the abdomen. The chance of a meaningful recovery was exceedingly slim. After a discussion with the patient and his family, comfort measures were instituted to allow natural death. He died 7 weeks after his initial presentation.

## Discussion

This patient was a 59-year-old man found on exploratory laparotomy to have a small bowel perforation secondary to metastatic disease of unknown origin. The pathology report revealed adenocarcinoma, the immunochemical characteristics of which suggested small bowel metastases from a primary lung cancer. However, the chest CT did not reveal a dominant mass. There were 2 small, noncalcified, nonspiculated pulmonary nodules, the largest measuring 1.1 cm, in the right upper lobe, and mildly enlarged hilar and subcarinal lymphadenopathy. Given this patient’s unusual presentation, the true nature of his malignancy required further evaluation. Positron emission tomography (PET)/CT and brain magnetic resonance imaging were ordered to complete his staging studies, as well as bronchoscopy to biopsy possible bronchial lesions or mediastinal lymph nodes. Lung cancer genetic mutation screening for known oncogenes—epidermal growth factor receptor (EGFR) mutation and anaplastic lymphoma kinase (ALK) rearrangements—were ordered to evaluate whether the tumor could be treated with tyrosine kinase inhibitors, such as erlotinib and crizotinib. In addition, there were plans to send the pathology specimens for gene expression profiling to confirm the likely source of malignancy. However, the patient died before these diagnostic tests could be performed. Although this presentation was distinctly unique for a lung primary, his smoking history, chest CT findings, and immunochemical profile taken together favor a lung primary.

Lung is one of the most common primary sites identified in patients who present with metastatic disease of unknown origin.^5,6^ In a review of 12 autopsy studies including 884 patients who succumb to CUP, the primary site was identified in 73% of patients, mostly in the lung (23%) and pancreas (24%) but also in the hepatobiliary tract (8%), kidney and adrenals (8%), colon (7%), genital system (7%), and stomach (6%).^4^ The most common site of metastases was lung (46%) followed by lymph node (35%), liver (23%), bone (17%), brain (16%), peritoneum (10%), and other uncommon organs (spleen, ovary, skin, soft tissue, parotid, thyroid, scalp, heart, and breast) accounting for the remaining 18% of cases.^4^ There were no documented cases of small bowel metastases in this large autopsy series of patients with CUP, connoting its rarity in our patient.

Smoking is a risk factor for CUP. In the European Prospective Investigation into Cancer and Nutrition cohort, a large ongoing multicenter, population-based prospective cohort (N = 476 940), smoking was identified as the most important risk factor in patients with CUP (relative risk [RR] = 3.22, 95% confidence interval [CI] = 2.24-5.97), especially in those who died within 12 months of diagnosis (RR = 5.12, 95% CI = 3.09-8.47). The authors of the study proposed that a substantial proportion of CUP may originate from smoking-related tumors. Waist circumference was also weakly correlated with slightly higher incidence of CUP (*P* < .01, RR = 1.29, 95% CI = 1.02-1.65).^3^ Our patient had a 12 pack-year smoking history, smoking a half pack of cigarettes per day since age 35. His normal weight before he became ill was reported to be 113.4 kg, and his height was 1.74 m, resulting in a calculated body mass index of 37, broadly classified as stage II obesity. His history of smoking combined with abdominal obesity were risk factors for developing CUP from a smoking-related tumor, such as lung adenocarcinoma.

Radiologic imaging studies can be useful in detecting the primary site. Retrospective studies show that PET detects unknown primary tumors in about 40% of patients with an overall 80.5% accuracy, 91.9% sensitivity, and 81.9% specificity. Lung was the most commonly detected primary tumor.^10,11^ However, a prospective study of CUP showed that integrated PET/CT did not demonstrate a significant diagnostic advantage over CT alone.^12^ The utility of these imaging techniques is limited as it is sometimes difficult to distinguish primary tumor from metastatic foci.^13^ Beyond imaging, immunohistochemical staining of metastatic tissue can be useful in suggesting the primary site.

When differentiating tumor origin, pathologists frequently exploit immunohistochemical profiles to achieve a tissue diagnosis.^14,15^ The basic immunohistochemistry panel delineates cancer cell lineage, using antibodies against leukocyte common antigen (LCA) for lymphoma, S100 and MART-1 for melanoma, cytokeratins such as AE1/AE3 and CAM5.2 for carcinoma, and vimentin for sarcoma. Vimentin may also be present in melanoma; however, the lack of other concomitant markers suggests sarcoma.^13^ Primary pulmonary tumors are commonly positive for markers such as TTF-1, napsin A, and CK7, but negative for CDX2 and CK20, which are considered gastrointestinal markers. Lung carcinoma typically has a CK7+/CK20− immunophenotype, while the opposite is expected of intestinal carcinoma, which generally stain CK7−/CK20+.^16^ In a pathology survey of 435 epithelial neoplasms, 100% of lung and 5% of colon adenocarcinomas were CK7+, while 10% of lung and 100% of colon adenocarcinomas were CK20+.^17^ However, primary rectum and small intestine tumors may lose CK20 expression and gain CK7 positivity.^18,19^ Hence, these markers must be used in combination with other markers specific for lung and intestinal tumors such as the transcription factors TTF-1 and CDX2.^15^

TTF-1 is classically considered the best marker of lung adenocarcinoma^20^ and effectively excludes squamous cell carcinoma.^21^ TTF-1 is a transcription factor that plays a role in embryologic thyroid and lung development.^20^ In the lungs, TTF-1 activates the expression of surfactant and clara cell secretory proteins by mediating the binding of corresponding promoter enhancers.^20^ Its restrictive expression in adults makes it a valuable marker for diagnosing tumors of lung origin.^22^ The percentage of positivity in pulmonary adenocarcinoma is approximately 75% to 80%.^15,23^ However, recent studies have shown infrequent TTF-1 expression in carcinomas arising from the colon and central nervous system,^23^ necessitating the use of other markers to improve specificity. Napsin A (novel aspartic proteinase of the pepsin family) is a novel marker for diagnosing pulmonary adenocarcinoma. In a retrospective review of 245 poorly differentiated non–small cell lung cancers, TTF-1 and napsin A were both specific (87% vs 100%), but TTF was more sensitive than napsin A (80 vs 64%) for lung adenocarcinoma.^24^ In this case report, the patient had tumor that was TTF-1 and napsin A positive, yet negative for intestinal (CK20 and CDX2) and neural (chromogranin, synaptophysin, and CD56) biomarkers, supporting metastatic adenocarcinoma with an immunoprofile favoring pulmonary origin.

The role of a limited panel of immunostains (CK7, CK20, TTF-1, CDX2) in highlighting lung primary was tested in a case series of 18 patients with primary lung cancer presenting with gastrointestinal involvement.^15^ The lung cancers were diagnosed on biopsies or surgical resections of the gastrointestinal tract. The immunohistochemistry of resected specimens exhibited tumor cells that were TTF-1 positive in 89% of cases and CK7+/CK20−/CDX2− in all cases. All of the cases had pulmonary nodules/masses radiologically documented; however, only a third of metastatic lesions were compared with their primary site of origin.^15^ Likewise, we were unable to compare both metastatic and primary lesions at histology in the reported case. However, the immunoprofile strongly suggested a pulmonary primary, and the history of smoking and positive lung findings on chest CT support the likelihood of a lung primary. Gene expression profiling may have provided a means by which to confirm or refute the results obtained from immunohistochemistry.

Gene expression profiling is emerging as a promising diagnostic tool for accurately predicting the primary tumor site in patients with CUP. The rationale for using molecular tumor profiling is that every metastatic tumor retains the basic genetic signature of its tissue of origin.^25^ Reverse transcriptase polymerase chain reaction (RT-PCR) and gene microarray are the 2 assays used in molecular tumor profiling and in establishing site-specific gene expression profiles. This diagnostic molecular technique was validated by identifying tissue of origin in primary tumors and metastatic carcinomas with known primary sites. Molecular tumor profiling correctly identified tissue of origin in 85% of carcinomas of known primary origin.^9^ Sensitivity for these assays range between 72% and 95%, while specificity approaches 99%.^6^ A recent, multicenter, blinded study by Handorf et al compared the diagnostic accuracy of gene expression profiling and immunohistochemistry for predicting primary site in a set of metastatic tumors from known primaries. The accuracy of these 2 molecular techniques was found to be similar, 89% for gene profiling compared with 83% for immunoprofiling (*P* = .013).^26^ In a similar study design, Weiss et al reported 79% accuracy for gene expression profiling compared with 69% for immunohistochemistry (*P* = .019), representing a modest improvement in diagnostic accuracy using gene expression profiling.^27^

Although validation is inherently difficult in patient with CUP, Greco et al demonstrated that 75% of latent primaries discovered months to years later were predicted by molecular tumor profiling. They found the diagnosis of primary site obtained by immunohistochemistry–matched gene expression profiling in 77% of cases.^28^ In a recent prospective trial by Hainsworth et al, molecular gene expression profiling was used to predict primary site and to guide site-specific therapy in patients with CUP. In their study, a 92-gene RT-PCR assay predicted tissue of origin in 247 of 252 (98%) patients. Furthermore, those patients who received site-specific therapy exhibited a survival advantage compared with previous data using empiric CUP regimens.^9^ The survival advantage was higher when predicting tumor types that are clinically more responsive to chemotherapy.^9^ Molecular gene expression profiling would have been a suitable method to confirm or refute the primary site in our patient. However, its impact on survival remains uncertain. The National Comprehensive Cancer Network currently does not recommend routine use of gene expression profiling in the workup of CUP as there is insufficient outcome data to support its routine use.

Gastrointestinal metastasis from lung cancer is a rare, yet well-documented clinical phenomena, with the small bowel representing the most common site of metastases to the gastrointestinal tract.^15,29-42^ The most common sites of lung metastases include regional lymph nodes (72% to 84%), liver (33% to 47%), bone (21% to 34%), brain (16% to 32%), and adrenals (20% to 29%),^40^ while the estimated incidence of gastrointestinal tract metastases from primary lung cancer range between 0.5% to 10%.^15^ The route of metastasis is not well characterized, but hematogenous spread has been hypothesized.^32,33,40^ On the other hand, histopathology from the patient in our case report revealed markedly distended lymphatics, engorged with tumor cells, suggesting prominent lymphatic involvement. Indeed, our patient exhibited extensive retroperitoneal as well as hilar and mediastinal lymphadenopathy.

In a large retrospective cohort study reviewing surgical pathology of 8159 patients with lung cancer, 21 had metastatic gastrointestinal involvement, comprising only 0.26% of their retrospective lung cancer cohort.^42^ Stomach and duodenal involvement typically caused gastrointestinal bleeding, while small intestine involvement caused obstruction and perforation. In the study, 6 of the 12 patients with lung adenocarcinoma metastasizing to the gastrointestinal tract had small bowel involvement presenting as either obstruction or perforation. Small bowel perforation cases were diagnosed at laparotomy after exhibiting signs and symptoms of an acute abdomen or peritonitis,^42^ resembling the clinical scenario in our case report.

Garwood et al reviewed the literature concerning bowel perforation from metastatic lung cancer and reported findings with multiple similarities to the presented case. The most common histologic type was adenocarcinoma, typically presenting as ulcerated masses with a predilection for the jejunum and ileum,^40^ consistent with the distribution of ulcerated masses in our patient. Perforated metastases were vastly more common in men (89% vs 11%) than women, and overall mean survival was 66 days after small bowel perforation.^40^ Our patient was male and died after 50 days. The most common location of primary lung tumor was right upper lobe,^40^ consistent with the location of lung nodules reported in our case. One study showed an upper lobe predominance of lung carcinoma in smokers,^43^ compatible with the hypothesis that the upper lobe masses in our patient were related to smoking.

Overall, the case presented in this report is consistent with the clinical and histopathological picture of bowel perforation secondary to metastatic lung cancer, although the absence of a dominant pulmonary mass in the presence of extensive metastatic small bowel involvement represents an unusual presentation of a relatively rare, yet well-documented clinical phenomenon. Given the lack of obvious primary lung cancer, molecular gene expression profiling and percutaneous transthoracic biopsy of the lung nodules would have been helpful in corroborating a lung primary.

## Conclusion

Discovery of metastatic adenocarcinoma of an unknown primary origin necessitates a careful clinical workup and complete histopathological examination, as well as concomitant imaging studies. However, preemptive diagnosis of small bowel involvement before perforation or obstruction is difficult, as the vast majority of patients initially present with an acute abdomen or peritonitis and often require emergency abdominal surgery. In this uncertain landscape, immunoprofiling is a valuable tool to determine tumor type and likely tissue of origin, which is most commonly adenocarcinoma and more often pulmonary in origin in smokers.
